# Depression Treatment Patterns among Elderly with Cancer

**DOI:** 10.1155/2012/676784

**Published:** 2012-08-27

**Authors:** Patricia A. Findley, Chan Shen, Usha Sambamoorthi

**Affiliations:** ^1^School of Social Work, Rutgers University, New Brunswick, NJ 08901, USA; ^2^Department of Biostatistics, MD Anderson Cancer Center, The University of Texas, Houston, TX 77030, USA; ^3^Department of Pharmaceutical Systems and Policy, School of Pharmacy, West Virginia University, Morgantown, WV 26506, USA; ^4^Department of Community Health and Preventive Medicine, Morehouse School of Medicine, Atlanta, GA 30310, USA

## Abstract

Little is known about cancer treatment patterns among the elderly as depression and cancer in this older population have not been well explored. This study seeks to fill a gap in the literature by using data from the Medicare Current Beneficiary Survey from years 2000–2005 to examine depression treatment patterns among elderly diagnosed with both cancer and depression. Depression treatments examined include antidepressants with and without psychotherapy. We found that of those with both cancer and depression, 57.7% reported antidepressant use only, 19.7% received psychotherapy with or without antidepressants, and 22.6% had no depression treatment. We found those with greater comorbidity, of a minority race, with lower levels of education, and living in rural areas were less likely to receive treatment for depression. These findings highlight the need to address disparities in the treatment of depression in the elderly population with cancer.

## 1. Introduction

Cancer among the elderly has grown in prevalence, in 2007, 70% of those diagnosed with cancer in the United States were over 65 years of age, with 44% over the age of 75 [1]. Currently, among the US population there are 7 million people over the age of 65 living with cancer [2]. Projections from 2010 to 2030 estimate a 67% increase in cancer incidence among the US population that is 65 years and older, compared to an increase of only 11% in younger age groups [3]. Given the rapid increases in the number of elderly diagnosed with cancer, greater awareness, identification, evaluation, and treatment of depression this group has gained attention [4]. However, the elderly are not been specifically studied in current cancer studies. For example, in a metareview of 100 studies, the prevalence of depression among individuals of all ages with cancer was reported to have reached as high as 38%–58%, but these studies did not include the elderly [5].

Although depression has been recognized as detrimental to cancer prognosis, treatment, and related quality of life, not much is known about the treatment of depression among elderly with cancer. In one study that included both elderly and nonelderly, using data from community oncology practices in the US, individuals over age 60 were less likely to receive antidepressants compared to younger individuals [6]. Furthermore, it has been reported that the elderly with cancer are at risk of developing subthreshold forms of depression, meaning depression may go unrecognized and untreated [7]. In fact, it has been reported that overall, the elderly as a subgroup are vulnerable to the undertreatment of depression [7]. Treating depression in elderly with cancer may help to prevent the adverse changes associated with depression such as decreases in quality of sleep, relationships, and quality of life, as well as increases in pain and other symptoms of cancer [4]. Such treatment of depression may support better treatment outcomes for elderly diagnosed with cancer.

The examination of existing data on elderly patients with cancer could lead to better treatment of depression in this growing population of elderly with cancer. Therefore, the primary objective of the present study is to estimate the rates of depression treatment and to identify subgroup differences in treatment (or lack of treatment) for depression among the elderly who are diagnosed with cancer and depression. This study uses cross-sectional data from multiple years of the Medicare Current Beneficiary Survey (MCBS), a nationally representative survey of Medicare beneficiaries.

This study was approved by the Institutional Review Board of Rutgers University.

## 2. Methods

### 2.1. Study Design

 This is a retrospective, cross-sectional study using data on the elderly derived from a nationally representative survey of Medicare beneficiaries. This is a descriptive study.

### 2.2. Data

Data for our study were obtained from the MCBS from 2000 to 2005. The MCBS is a rotating panel survey of a nationally representative sample of Medicare beneficiaries [8]. Survey data are collected by using in-depth computer-assisted personal interviews (CAPI) and are conducted every four months to capture healthcare utilization (including prescriptions filled) and non-Medicare payment sources reported by respondents. The MCBS provides both cross-sectional and longitudinal data on the health status, treatment, Medicare and non-Medicare utilization, prescription drug use, and expenditures of Medicare beneficiaries. These data also provide socioeconomic and demographic characteristics of the beneficiaries. The current study used the annual data from the calendar year 2000–2005 “Cost and Use” files.

### 2.3. Identification of Cancer

We identified individuals with cancer based on their responses to two survey questions in the MCBS. Survey respondents were asked whether “a doctor (ever) told (you) that (you) had any (other) kind of cancer, malignancy, or tumor other than skin cancer?” A similar question was asked about any diagnosis of skin cancer. We defined individuals with cancer based on an affirmative response to one or both of the questions. We included all cancers as the emotional response to a cancer diagnosis can vary by individual despite the type of cancer.

### 2.4. Identification of Diagnosed Depression

Diagnosed depression was identified using fee-for-service claims of the Medicare beneficiaries and based on the *International Classification of Diseases, 9th Edition, Clinical Modification* (ICD-9-CM) codes [9]. The ICD-9-CM codes we used were 296.2 (major depressive disorder, single episode), 296.3 (major depressive disorder, recurrent episode), 300.4 (neurotic depression), 309.1 (prolonged depressive reaction), and 311 (depression, not elsewhere classified). These codes were used to identify diagnosed depression in a prior published study [10]. 

### 2.5. Analytical Sample

 Among the elderly with cancer and depression, we excluded beneficiaries who were not enrolled in Medicare throughout the entire calendar year to ensure we had complete annual data. We excluded those who were institutionalized (i.e., in a nursing home), who were enrolled in Medicare managed care, and who died during the observation years. We used only fully enrolled beneficiaries to ensure a uniform observation period. Since we derived psychotherapy use from claims, Medicare-managed enrollees were excluded because claims were not available for those individuals. Thus, the final sample for analysis consisted of data for 865 elderly Medicare beneficiaries who had both cancer and depression in the years from 2000 to 2005. 

## 3. Measures

### 3.1. Dependent Variable 

#### 3.1.1. Depression Treatment: Antidepressant Use

 We derived antidepressant use from self-reported prescription drug data. We could not use pharmacy claims because Medicare Part D was not enacted until 2006. For the MCBS, to minimize recall errors in medication use, a number of steps had been taken, including holding interviews at 4-month intervals, asking the survey respondents to bring prescription drug bottles to the interview, and providing the respondents with calendars on which to record their use of prescription drugs. We used drug names to identify antidepressants. Drugs included in the class of antidepressants were selective serotonin reuptake inhibitors (SSRI), serotonin/norepinephrine reuptake inhibitors (SNRI), tricyclics (TCA), tetracyclics, and monoamine oxidase inhibitors (MAOI) [11]. 

#### 3.1.2. Treatment of Depression: Psychotherapy

We identified psychotherapy treatment from Medicare claims based on current procedural terminology (CPT) codes used in previous published analyses [12]. We categorized depression treatment patterns into three groups based on the use of antidepressants and receipt of psychotherapy: (1) no depression treatment; (2) antidepressant use only; (3) psychotherapy use with or without antidepressants. We did not distinguish psychotherapy with and without antidepressants because most of the people who received psychotherapy also reported antidepressant use, with less than 5% (*n* = 46) receiving psychotherapy only. 

### 3.2. Independent Variables

Independent variables consisted of demographic characteristics: gender (male, female), race (white, minority), age (65–74 years, 75 and older), marital status (married, widowed, and other), and geographic region (urban/rural). Socioeconomic status consisted of education (less than high school, completed high school, some college, completed college, and above), poverty status (<200% of the poverty level, ≥200%), and access to care, which was measured with an indicator variable (yes, no) for prescription drug coverage. As noted previously, because Medicare Part D did not start until January 2006, we constructed a variable on the use of prescription drugs using the same method reported in published studies [13, 14]. We measured health status with a number of variables: general health perception (excellent/very good, good, and fair/poor); number of chronic medical conditions, which included arthritis, diabetes, heart disease, hypertension, stroke, respiratory conditions, and/or osteoporosis; difficulty with activities of daily living (ADLs) based on the number of ADL categories that were considered difficult (none, 1-2, and 3–6). We assessed lifestyle risk factors in two areas: body mass index (BMI) and smoking status. A BMI of less than 25 kg/m^2^ indicates the individual is underweight or normal; a BMI in the range from 25 to 29.9 kg/m^2^ indicates the individual is overweight; a BMI greater than 30 kg/m^2^ indicates the individual is obese or morbidly obese [15]. We used three categories for smoking status (current smoker, past smoker, and never smoked). In multinomial logistic regressions, we combined the categories of current smoker and past smoker.

### 3.3. Statistical Techniques

We conducted both bivariate and multivariate analyses, and tested the group differences in depression treatment using chi-squared statistics. We employed multinomial logistic regression on the depression treatment categories to examine treatment patterns. From multinomial logistic regression, we transformed the parameter estimates to odds ratios and reported their corresponding 95% confidence intervals. In the multinomial regression, for the dependent variable, the reference group was “no depression treatment.” We accounted for the complex design of the MCBS by conducting all these analyses with survey procedures in SAS 9.2 [16].

## 4. Findings

We found that 5% of the elderly with cancer were diagnosed with depression. This percentage remained consistent for all the years studied (data not shown but available upon request). A description of this study sample of 865 elderly Medicare beneficiaries with depression and cancer is provided in Table 1. The majority of beneficiaries in our sample were white women who lived in urban areas. About half (48%) of them had low income as measured by an annual income of less than 200% of the poverty threshold. Thirty-six percent had completed a high school education. Common physical conditions (not presented in tabular form) included arthritis (77%), hypertension (66%), and heart disease (47%). Forty-four percent had difficulty with at least one ADL.

In our sample, of those who reported depression, 57.7% were prescribed antidepressants *only*, 19.7% had psychotherapy with or without antidepressants, and 22.6% had no depression treatment (Table 2). Among the significant findings (.01 < *P* < .05), we found that a greater proportion of the elderly living in rural areas had no depression treatment compared to those living in urban areas (29.2% versus 20.6%). Compared to the elderly with less formal education, a higher proportion of the elderly with a college education received psychotherapy treatment of depression and a lower proportion had no treatment. For example, 13.5% of those with less than a high school education received psychotherapy compared with 28.2% of those who completed college; 25.6% of the elderly who did not complete high school had no depression treatment versus 17.2% in the group who completed college. 

Table 3 summarizes findings from the multinomial logistic regression on depression treatment. In this analysis “no treatment” served as the reference group for the dependent variable, depression treatment. We assessed the fit of our model by evaluating the overall model and statistically testing the contribution of each of the independent variables. In terms of overall model evaluation, we tested the fit of the final model against a null or intercept-only model. We found that all tests (likelihood ratio, score, and Wald) were statistically significant at *P* < .0001 suggesting that at least one of the independent variables was associated with depression treatment categories. We also tested parameter estimates of each independent variable using Wald chi-square tests. Based on these tests, we observed that gender, female, and community status were significantly associated with depression treatment categories. None of the other variables were statistically significant. In terms of individual categories, nonwhites were less likely to receive antidepressants only compared to no treatment at all (adjusted odds ratio [AOR] 0.54; 95% confidence interval [CI] = 0.30–0.95). Those living in urban areas were more likely to receive antidepressants rather than no treatment (AOR 1.52; 95% CI = 1.04–2.23) and those with less than a high school education or at most a high school education were less likely to receive psychotherapy ([AOR 0.36; 95% CI = 0.15–0.83] and [AOR 0.43; 95% CI = 0.20–0.93], resp.). The elderly who had more chronic conditions were more likely to receive antidepressants only compared to no treatment (AOR 1.17; 95% CI = 1.00–1.36). Conversely, the elderly with one to two areas of impairment in activities of daily living were less likely to receive antidepressants (AOR 0.65; 95% CI = 0.43–0.99). All of these findings were statistically significant at the .01 < *P* < .05 level.

## 5. Discussion

To examine patterns of depression treatment among elderly Medicare beneficiaries with a diagnosis for both cancer and depression, we used data from the MCBS, a nationally representative survey of individuals enrolled in Medicare in years from 2000 to 2005. We found that 24% of our study sample received no treatment of their depression, whereas an overwhelming majority (76%) reported using antidepressants and/or psychotherapy. Unfortunately, there are no published studies with which to compare the findings of the current study. For the purpose of discussion, we compare our findings to studies conducted in the general population (i.e., included nonelderly).

When examining the type of treatment, only a minority (19.7%) of the elderly received psychotherapy with or without antidepressant medications. Fifty-seven percent of our sample reported using antidepressant medications *only*. This result is similar to what Akincigil et al. reported in their study using MCBS data from 2005 where 67.3% of the general elderly population was using antidepressants and 14.3% used psychotherapy [17]. These findings were not surprising given the current trends in the treatment for depression across the nation overall where there has been a shift toward an increased reliance on medications over psychotherapy in all age groups. This is evidenced in a longitudinal study of the general population using Medical Expenditure Panel Survey (MEPS) data from 1996 to 2005. In this study, Mackenzic et al. [18] found that among elderly with depression, rates of antidepressant use increased between 1996 and 2005, whereas the rates of psychotherapy decreased. Wei et al. also found the use of psychotherapy to be uncommon among the elderly diagnosed with depression, despite its acknowledged efficacy [12]. We suggest that the limited use of psychotherapy may be attributed to several factors, including help-seeking behavior among the elderly [18], racial or ethnic disparities in health care [19, 20], restricted access to specialty mental health providers [21], low cultural acceptance of psychotherapy as a treatment [22], and the high cost of psychotherapy [23]. 

We found a lack of depression treatment for some of the elderly in our study. The lack of treatment for depression in individuals with cancer might be related to the competing demands of healthcare management and the prioritization of treatment of cancer with other chronic physical conditions the individual may be experiencing [24]. It is important to note that we did not know the date of diagnosis. Therefore, we could not distinguish between the elderly who had completed cancer treatment and those who were actively receiving cancer treatment. Active cancer treatment would likely supersede treatment for depression, in particular because these patients would likely be seen by oncologists rather than primary care physicians who might provide more inclusive care [25]. Furthermore, although cancer treatment itself is known to induce depression, efforts to manage pain and fatigue might play a more prominent role over the treatment for depression [26]. It has been reported that prevalence of fatigue is greater than 50–70% in advanced cancer patients and the prevalence of pain is 80% in elderly patients with advanced cancer [27]. Therefore, the complexities of treating a serious chronic disease and the coordination of the medical care required to address the comorbid conditions may lead to a lower priority for the treatment for depression [28]. Additionally, detecting depression in an elderly cancer patient may require special consideration to address agedrelated issues such a memory loss. Traeger and Pirl recommend adapting standard depression assessments by adding additional questions that characterize functioning (e.g., tell me about a typical day) rather than relying on specific recall [29]. These adaptations allow the physician to gather pieces of necessary information to see if the patient meets the criteria for depression as defined by the Diagnostic and Statistical Manual of Mental Disorders-IV-TR [30].

Our findings with regard to racial differences in depression treatment are consistent with the national treatment patterns for the general population. Another study has reported increased depression among the minority elderly with chronic illnesses [31]. However, when analyzing the trends in antidepressant use among individuals, regardless of a diagnosis for depression, and based on national data comparing the trends in 1996 to those in 2005, Olfson and Marcus [32] found that increases in antidepressant use were evident across all racial groups examined, except for among African Americans, who had comparatively low rates of use in both years (1996, 3.61%; 2005, 4.51%). Akincigil et al. [17] found an uneven trend in racial differences in antidepressant use from 1992 to 2005 for the elderly in their study. Their study showed that all minorities received less antidepressant treatment compared to whites in 2002–2005, which is similar to our findings in our study period of 2000–2005.

With respect to geographic area, we found the rates of psychotherapy use were different between urban and rural areas, with those in urban areas receiving more treatment. This is inconsistent with a recent prospective study on a small sample that found no widespread differences between cancer survivors living in rural versus nonrural areas with respect to mental health resource use [22]. These authors suggested that some of the decreased use of mental health resources in rural areas may be due to poorer accessibility and availability of mental health professionals in those communities. It has also been reported that the elderly living in rural areas may rely on their religion more than formal mental health services to support them when depressed [33]. 

We found that education level also influenced the likelihood of receiving psychotherapy. Individuals who had attained a higher level of education (i.e., college) were more likely to receive psychotherapy compared to those who had attained only a high school education or less. This was not surprising. Other researchers have found that higher levels of education influence more positive attitudes toward the acceptance of mental health services, particularly for men [18].

This study has several strengths worth noting. We used a nationally representative data set on a large number of Medicare beneficiaries with cancer that includes comprehensive information obtained from the survey and Medicare claims. We included a number of relevant covariates and use of a case-finding algorithm for quality measures to identify depression [34]. We included the use of psychotherapy, a variable that is not usually analyzed in studies of the elderly with cancer. 

Despite the noted strengths, our study findings need to be interpreted in the context of their limitations. With respect to selection bias, by focusing on diagnosed depression, we may have excluded individuals who were not diagnosed but were given treatment; thus, our treatment rates may be underestimated. We also identify those individuals who were first diagnosed for depression then subsequently treated for depression. Medicare-managed care enrollees were excluded; thus, generalizability is limited. Although we pooled multiple years, the sample size was too small to allow us to separately analyze psychotherapy without antidepressants, which may have implications for individuals with cancer who are undergoing treatment. 

We did have a limitation with prescription benefit coverage. As prescription benefits under Medicare Part *D* were not implemented until 2006, the year after our study period; prescription drug use was self-reported. Self-reporting has been shown to be susceptible to underreporting [35]. Although efforts were taken to minimize bias in the self-reported pharmacy data, preliminary results from a validation study conducted by the Centers for Medicare and Medicaid Services (CMS) suggest that there is an underreporting rate of approximately 15–18% in the MCBS [35]. However, these data are heavily used in published studies of depression among many different types of populations [14, 36].

We could not control for clinical factors such as the specific type of cancer or duration and stage of cancer, which may be associated with the likelihood for depression treatment. However, by excluding individuals who died during the same calendar year for which they were included in the data set, we were able to control for end-stage cancer. By focusing on diagnosed depression, we may have excluded individuals who were not diagnosed but were given treatment; thus, our treatment rates may be underestimated. Medicare-managed care enrollees were excluded; thus, generalizability is limited. Although we pooled multiple years, the sample size was too small to allow us to separately analyze psychotherapy without antidepressants, which may have implications for individuals with cancer who are undergoing treatment.

Another area of limitation that is not restricted to our study alone is the identification of depression by ICD-9-CM codes. Although this is an accepted method in numerous publications [37–40], relying on these codes results in a very heterogeneous population since the codes were creating for billing purposes and not for treatment purposes. Future research on smaller populations using prospective studies and/or chart reviews might gather qualitative data more representative of individuals with depression and demonstrate the nuances of treatment decision making. 

Despite these limitations, our study adds to the nascent literature on cancer and depression treatment in the elderly. The treatment for depression remains a concern because of the impact of depression on the success of cancer treatment [4] and the individual's overall prognosis. As proposed by the Institute of Medicine, survivorship care plans for cancer survivors include treatment for depression [41]. Our findings suggest that providers and policy makers need to address the barriers to psychotherapy use for the elderly, particularly in rural areas. Furthermore, interventions should be developed to educate individuals with cancer on the role of depression in their overall health care needs. Such interventions should target minority racial groups and cancer survivors living outside of urban areas to promote the identification and evaluation of depression and the appropriate treatment of depression among the elderly with cancer. 

## Figures and Tables

**Table 1:** Description of study sample with reported cancer and diagnosed depression. Medicare Current Beneficiary Survey, 2000–2005.

	*N*	Weighted %
Year		
2000	125	13.1
2001	127	14.3
2002	170	18.7
2003	158	18.9
2004	147	18.2
2005	138	16.8
Gender		
Female	593	69.5
Male	272	30.5
Race/ethnicity		
White	783	90.5
African American	27	3.0
Latino	32	4.1
Other	23	2.4
Age in years		
65–69	113	14.2
70–74	183	25.0
75–79	202	26.9
80+	367	33.9
Marital status		
Married	382	45.5
Widowed	370	41.5
Divorced/separated	87	10.6
Other	26	2.5
Community status		
Urban	635	76.7
Rural	230	23.3
Education		
Less than high school	221	25.6
High school	308	36.1
Some college	146	16.9
College	186	21.4
Poverty status		
LT 200%	427	47.8
≥200%	438	52.2
Prescription drug coverage		
Yes	683	80.1
No	182	19.9
Health status		
Excellent/very good	64	7.0
Good	191	22.6
Fair/poor	107	12.5
Functional status (ADL)		
No difficulties with ADLs	478	55.9
1-2	236	27.0
3–6	149	17.1
Smoking status		
Current smoker	73	9.0
Past smoker	452	52.7
Never smoked	340	38.2
Body mass index		
Underweight/normal	383	42.1
Overweight	308	35.9
Obese	168	22.0

Note: Based on 865 person—years of elderly Medicare beneficiaries with reported cancer and diagnosed depression.

ADL: activity of daily living.

**Table 2:** Number and weighted percent of study sample by depression treatment. Medicare Current Beneficiary Survey, 2000–2005.

ALL	AD only		Psyc w/o AD		No treatment		
	*N*	Wt%	*N*	Wt%	*N*	Wt%	Sig.
	493	57.7	168	19.7	204	22.6	
Year							
2000	75	58.9	21	18.5	29	22.6	
2001	75	58.6	23	20.1	29	21.3	
2002	81	46.5	39	23.8	50	29.7	
2003	100	63.7	31	19.4	27	16.9	
2004	89	63.7	24	15.7	34	20.6	
2005	73	55.0	30	20.7	35	24.3	
Gender							
Female	329	56.3	130	22.4	134	21.3	
Male	164	60.7	38	13.8	70	25.5	
Race							
White	453	58.2	151	19.9	179	22.0	
Minorities	40	53.0	17	18.6	25	28.4	
Age in years							
65–74	171	58.9	65	21.7	60	19.4	
75 and older	322	56.9	103	18.5	144	24.6	
Marital status							
Married	221	57.6	61	16.8	100	25.6	
Widowed	210	58.3	77	21.0	83	20.7	
Other	62	55.7	30	26.2	21	18.1	
Community status							*
Urban	366	58.5	132	20.9	137	20.6	
Rural	127	54.8	36	16.0	67	29.2	
Education							*
LT HS	130	60.9	32	13.5	59	25.6	
HS	185	60.5	52	17.0	71	22.5	
Some college	73	50.8	35	24.7	38	24.5	
College	104	54.7	49	28.2	33	17.2	
Poverty status							
LT 200%	254	61.2	68	16.0	105	22.8	
≥200%	239	54.4	100	23.2	99	22.4	
Prescription drug coverage							
Yes	393	58.2	133	19.7	157	22.1	
No	100	55.3	35	20.1	47	24.6	
Health status							
Excellent/very good	141	55.4	49	20.0	65	24.7	
Good	154	59.7	50	19.6	59	20.8	
Fair/poor	195	57.5	69	19.9	80	22.6	
Functional status (ADL)							
No difficulties	275	57.5	94	20.5	109	22.0	
1-2 ADLs	125	53.4	49	21.0	62	25.6	
3–6 ADLs	91	63.5	25	15.9	33	20.5	
Smoking status							
Current smoker	43	59.6	12	16.7	18	23.7	
Past smoker	253	56.8	88	20.9	111	22.3	
Never smoked	197	58.4	68	18.9	75	22.7	
Body mass index							
Und/normal	202	52.5	79	22.2	102	25.3	
Overweight	177	58.6	60	18.7	71	22.7	
Obese	109	64.3	28	17.3	31	18.5	
Number of chronic conditions							
0–2	201	54.9	67	18.7	101	26.4	
Three or more	292	59.7	101	20.5	103	19.8	

Note: Based on 865 person—years of elderly Medicare beneficiaries with cancer and depression using Medicare Current Beneficiary Survey 2000–2005. Asterisks represent significant group differences by depression treatment based on chi-squared tests.

AD: antidepressants; Psyc w/o: psychotherapy use with or without antidepressants; Sig: significance; Wt: weighted; ADL: activity of daily living; Und: underweight; HS: high school; LT: less than.

*.01 ≤ *P* < .05 [Note: only a single asterisk appears in the table].

**Table 3:** Adjusted odds ratios and 95% confidence intervals from multinomial logistic regression on depression treatment. Medicare Current Beneficiary Survey, 2000–2005.

	Antidepressant only			Psychotherapy with or without antidepressants		
	AOR	95% CI	Sig.	AOR	95% CI	Sig.
Year						
2000						
2001	1.01	[0.55, 1.85]		1.05	[0.48, 2.30]	
2002	0.56	[0.30, 1.04]		0.87	[0.42, 1.80]	
2003	1.38	[0.74, 2.57]		1.38	[0.60, 3.17]	
2004	1.14	[0.61, 2.15]		0.92	[0.40, 2.13]	
2005	0.79	[0.46, 1.35]		0.95	[0.44, 2.08]	
Gender						
Female	0.99	[0.58, 1.68]		1.95	[0.99, 3.84]	
Male						
Race						
White						
Minorities	0.54	[0.30, 0.95]	*	0.67	[0.26, 1.69]	
Age in years						
65–74						
75,+	0.83	[0.49, 1.41]		0.65	[0.35, 1.23]	
Marital status						
Married						
Widowed	1.39	[0.84, 2.28]		1.69	[0.85, 3.37]	
Other	1.20	[0.58, 2.49]		1.86	[0.74, 4.70]	
Community status						
Urban	1.52	[1.04, 2.23]	*	1.80	[0.98, 3.33]	
Rural						
Education						
LT HS	0.69	[0.35, 1.34]		0.36	[0.15, 0.83]	*
HS	0.78	[0.40, 1.51]		0.43	[0.20, 0.93]	*
Some college	0.70	[0.37, 1.34]		0.63	[0.31, 1.30]	
College						
Poverty status						
LT 200%	1.12	[0.74, 1.70]		0.73	[0.41, 1.30]	
≥200%						
Prescription drug coverage						
Yes						
No	0.94	[0.60, 1.46]		1.16	[0.63, 2.15]	
Health status						
Excellent/very good						
Good	1.27	[0.78, 2.07]		1.25	[0.74, 2.10]	
Fair/Poor	1.06	[0.65, 1.72]		1.37	[0.70, 2.65]	
Number of chronic conditions						
	1.17	[1.00, 1.36]	*	1.16	[0.94, 1.41]	
Functional status (ADL)						
No difficulties						
1-2 ADLs	0.65	[0.43, 0.99]	*	0.72	[0.40, 1.30]	
3–6 ADLs	0.95	[0.52, 1.72]		0.67	[0.34, 1.33]	
Smoking status						
Current smoker	1.00	[0.63, 1.60]		1.13	[0.64, 2.01]	
Other						
Body mass index						
Under/normal						
Overweight	1.26	[0.81, 1.95]		1.10	[0.65, 1.87]	
Obese	1.63	[0.90, 2.95]		1.02	[0.45, 2.32]	
Overall model evaluation						
Test		Chi-square	DF	*P*-value		

*Likelihood ratio*		291214.347	48	<.0001		
*Score *		283025.448	48	<.0001		
*Wald*		138.4648	48	<.001		

Note: Based on 865 person—years of elderly Medicare beneficiaries with cancer and depression using Medicare Current Beneficiary Survey 2000–2005. The regression also included intercepts. The reference group for the dependent variable was “No depression treatment.” Asterisks represent significant differences in likelihood of depression treatment compared to the reference group based on multinomial logistic regression.

AOR: adjusted odds ratio; CI: confidence interval; Sig: significance; LT: less than; HS: high school; high school; ADL: activity of daily living.

*.01 ≤ *P* < .05.
